# Linkage to Care, Retention in Care and Treatment Uptake Among Patients Diagnosed With Chronic Hepatitis B in Norway, 2008–2022

**DOI:** 10.1111/jvh.70089

**Published:** 2025-09-22

**Authors:** Beatriz Valcarcel Salamanca, Asgeir Johannessen, Olav Dalgard, Robert Whittaker

**Affiliations:** ^1^ Department of Infection Control and Vaccines Norwegian Institute of Public Health Oslo Norway; ^2^ Department of Infectious Diseases Vestfold Hospital Trust Tønsberg Norway; ^3^ Institute of Clinical Medicine University of Oslo Oslo Norway; ^4^ Department of Infectious Diseases Akershus University Hospital Lørenskog Norway

**Keywords:** chronic hepatitis B, disease elimination, electronic health records, Norway, public health surveillance

## Abstract

People living with chronic hepatitis B infection (PLWHB) need life‐long care to monitor liver health and treatment need. Data on clinical follow‐up for PLWHB are essential to monitor the health system response to this infection. We used linked national registry data to calculate the proportion of diagnosed PLWHB in Norway linked to specialist care (LTC), treated and retained in specialist or primary care (RIC) from 2008 to 2022. We described the outcomes by time, age, sex, region of residence, place of birth and residence status. Using log‐binomial regression, we explored how these factors were associated with ever being LTC and being RIC during the last 12 months of the study period. Among 10,542 diagnosed PLWHB, 8301 (79%) had ever been LTC and 2454 (23%) had received treatment. In the first 2 years after LTC, 64% were still RIC. At the end of the study period, 4476 (50%) of 8979 PLWHB still resident in Norway had been RIC in the last 12 months. PLWHB born outside Norway had a higher probability of LTC (relative risk [RR]: 1.24; 95% confidence interval [CI] 1.19–1.29) and RIC (RR: 1.67; 95% CI 1.53–1.84). Other significant associations with smaller effect sizes included a higher probability of LTC among PLWHB aged < 25 years and a lower probability of RIC when diagnosed from 2010 to 2013 or aged ≥ 65 years. The management of diagnosed PLWHB in Norway is suboptimal. Our study provides a framework for how key performance indicators can be monitored in ongoing national surveillance.

## 1 Introduction

Chronic infection with hepatitis B virus (CHB) causes a notable global public health burden, with an estimated 254 million prevalent infections and 1.1 million deaths in 2022 [1]. The World Health Organisation (WHO) aims to eliminate hepatitis B as a public health threat by 2030 [2]. In Norway, the prevalence of CHB is low in the general population (< 0.5%) and > 95% of new diagnoses are among immigrants infected before migration [3]. Similarly concentrated epidemics are observed in many other countries of the European Union (EU) and European Economic Area (EEA) [4].

Regular clinical follow‐up of people living with hepatitis B (PLWHB) is essential to determine the phenotype of the infection, inform the need for treatment, and prevent onward transmission and severe liver disease. In Norway, all testing and clinical follow‐up for CHB is state‐funded and available at no cost, including to undocumented migrants. Clinicians in Norway have historically been advised to follow guidelines from the European Association for the Study of the Liver [5, 6]. National guidelines were first published in 2017 [7]. Following diagnosis in primary or specialist healthcare, the standard of care is referral of all PLWHB to specialist care at hospital outpatient clinics for initial evaluation (i.e., linkage to care). Follow‐up thereafter depends on the disease phenotype and clinical progression. PLWHB are recommended to be followed up with a blood test at a specialist outpatient consultation at least annually, although persistent hepatitis B e‐antigen (HBeAg) negative chronic infection (‘inactive carriers’) may be referred back to their general practitioner for annual follow‐up [8]. When the national guidelines were revised in 2022, treatment eligibility criteria were expanded (specifically, the alanine transaminase [ALT] level indicating antiviral treatment was reduced from 80 to 40 U/L for patients with mild or no fibrosis [8]).

Data on different stages of clinical follow‐up for CHB are essential to monitor the health system response and progress towards elimination. These include indicators for the proportion of diagnosed PLWHB linked to care for initial evaluation (LTC), retained in care (RIC) and on treatment [9]. Yet, how well PLWHB are connected to care has been understudied globally [10]. In Norway, limited data on these indicators are available [11], a tendency seen across EU/EEA countries [9]. We used linked national registry data to calculate LTC, RIC and treatment uptake among diagnosed PLWHB in Norway from 2008 to 2022, and studied demographic factors associated with these outcomes.

## 2 Materials and Methods

### 2.1 Study Population

Our study population was patients diagnosed with CHB in Norway from January 2008 to December 2022. We used two data sources to identify this study population:

1. The Norwegian Surveillance System for Communicable Diseases (MSIS), which contains data on notified patients diagnosed with CHB infection, is defined as the presence of hepatitis B virus surface antigen (HBsAg) in the blood for ≥ 6 months.
2. The Norwegian Patient Registry (NPR), which contains data on all inpatient and outpatient hospital stays in Norway, states that all newly diagnosed PLWHB should be referred to specialist care and therefore registered in NPR soon after initial diagnosis.

We used these two data sources to identify patients diagnosed with CHB in Norway because national identity numbers were necessary to link our study population to other registries to study our outcomes, but the completeness of data on national identity numbers in MSIS was low in some years (< 80% prior to 2017). The study period was restricted to 2008 onwards owing to the low completeness of personal identifiers in MSIS, and that identity numbers were not collected in NPR before that year.

From MSIS we included notified patients diagnosed with CHB with a linkable personal identifier (MSIS cohort). From NPR we identified patients who were not in the MSIS cohort, but had the International Classification of Diseases, 10th revision (ICD‐10) code B18.0 or B18.1 (CHB with/without hepatitis D) registered for at least two stays in a somatic hospital (NPR cohort). We limited our NPR cohort to patients registered with B18.0 or B18.1 at least twice to better ensure we identified true CHB diagnoses and not miscoding (e.g., B18.0 or B18.1 given to patients with chronic hepatitis C [B18.2] or patients with acute or resolved hepatitis B virus infection). We used the date of first CHB consultation in NPR as a proxy for ‘diagnosis’ date. A comparison of how well first CHB consultation dates in NPR reflect diagnosis dates in MSIS is presented in Appendix S1: part 1.

Data from MSIS and NPR included diagnosis date/consultation date, age, sex and county of residence. We supplemented this with data from the National Population Register on residence status at the end of 2022 (e.g., resident in Norway, out‐migrated or died) and country of birth.

### 2.2 Outcomes

Our outcomes were LTC (ever), RIC (in the first 3 years after initial LTC and in the last 12 months of the study period) and treatment uptake (ever and in the last 12 months of the study period). We defined our outcomes using data from NPR, the Norwegian Registry for Primary Health Care (NRPHC) and the Norwegian Prescribed Drug Registry (NPDR). NRPHC contains data from all municipalities on healthcare contacts in primary care. Personal identifiers are available from July 2016. NPDR contains data on medicines dispensed by prescription from pharmacies in Norway since 2004.

Table 1 presents the data sources, codes, time points and cohorts used to define each outcome. We had access to all instances where the codes listed were registered. For all outcomes, we analysed the proportion and included data until the end of June 2023, that is, a minimum of 6 months of follow‐up. Thus, the ‘last 12 months of the study period’ refers to July 2022–June 2023. Data beyond June 2023 were not available at the time of data extraction.

**TABLE 1:** Data sources, codes and time points used to define study outcomes.

Outcome	Data source and definition	Time points	Cohort
Linkage to care	Norwegian Patient Registry: First‐time registration of ICD‐10 codes for CHB (B18.0 or B18.1) for an outpatient hospital consultation in medical (code 30–39) or paediatric (code 44) departments^a^, OR Norwegian Prescribed Drug Registry: First‐time registration of one of the following ATC codes: antiviral treatment (J05AF05, J05AF07, J05AF08, J05AF10, J05AF11 and J05AF13) or peg‐interferon alpha 2a/2b (L03AB10 and L03AB11)^b^.	Ever during the study period.	Whole study population
Retention in care	Norwegian Patient Registry: Same definition as for linkage to care, OR Norwegian Prescribed Drug Registry: Same definition as for linkage to care, OR Norwegian Registry for Primary Health Care: Registration of the ICPC‐2 code for viral hepatitis (D72) for a visit to a general practitioner.	In the following time windows after the initial linkage to care: 3–11 months (1st year)^c^ 12–23 months (2nd year) 24–35 months (3rd year)	Whole study population
		Within the last 12 months^d^ of the study period (i.e., still retained in care).	Patients still resident in Norway at the end of 2022
Treatment uptake	Norwegian Prescribed Drug Registry: Same definition as for linkage to care.	Ever during the study period.	Whole study population
		Within the last 12 months^d^ of the study period (i.e., still on treatment).	Patients still resident in Norway at the end of 2022

Abbreviations: ATC, Anatomical Therapeutic Chemical; ICD‐10, International Classification of Diseases, 10th revision; ICPC‐2, International Classification for Primary Care, 2nd edition.

^a^ Department codes are reported according to the Norwegian IK‐44/89 coding system.

^b^ In Norway, patients are preferentially treated with nucleoside/nucleotide analogues, such as tenofovir disoproxil fumarate (J05AF07), tenofovir alafenamide (J05AF13) and entecavir (J05AF10). Treatment with nucleoside/nucleotide analogues is generally lifelong. A 48‐week course of pegylated‐interferon (L03AB10 and L03AB11) is primarily reserved for patients with a high chance of treatment success, such as young HBeAg positive patients with a genotype A or B infection [8].

^c^ We excluded follow‐up 0–2 months after initial linkage to care, considered to be related to the initial linkage.

^d^ We had follow‐up data until June 2023, so the last 12 months refers to July 2022–June 2023.

For the MSIS cohort, we also analysed the median number of months from diagnosis to LTC, with the interquartile range (IQR). We could not analyse time to LTC for the NPR cohort, as we did not have data on the date of diagnosis for these patients.

### 2.3 Data Linkage and Analysis

We linked data from all registries together using a deidentified project‐specific number, based on national identifiers, either the Norwegian national identity number (‘Fødselsnummer’) or D number for temporary residents.

We described our outcomes (Table 1) by data source, the codes registered, time, age, sex, region of residence, place of birth and residence status. We conducted log‐binomial regression to identify factors associated with ever being LTC and RIC in the last 12 months of the study period. For LTC, we included the covariates year of diagnosis, age at diagnosis, region of residence at diagnosis, sex, place of birth and residence status. For RIC in the last 12 months, we limited the cohort to diagnosed PLWHB who were registered as still resident in Norway and included the covariates year of diagnosis, age as of the end of the study period, region as of the end of the study period, sex and place of birth. For all models, univariable analyses were initially performed. Independent variables with a *p* ≤ 0.1 were included in multivariable models. Measures of association were expressed as relative risks (RR) with 95% confidence intervals (CI). For each outcome, we also conducted sensitivity analyses using different models, cohorts, outcome definitions or input data to explore how these affected our results.

As we lacked data on clinical indicators for treatment (liver stiffness, hepatitis B virus DNA levels, HBeAg status and ALT levels), we did not conduct further analysis on treatment outcomes.

All data analyses were performed using R (version 4.0; R Foundation for Statistical Computing, Vienna, Austria). A *p*‐value < 0.05 was considered statistically significant. No Artificial Intelligence Generated Content tools were used in the writing of this manuscript.

## 3 Results

### 3.1 Study Cohort

We identified 10,542 diagnosed PLWHB from January 2008 to December 2022. Characteristics of these patients are presented in Table 2. The median age at diagnosis was 36 years (IQR: 29–47), 58% were male, 86% were born outside Norway and 65% were residents in Oslo or South‐east Norway. Equivalent tables by data source used to identify the study population (MSIS, *n* = 5726, 54%; NPR, *n* = 4816, 46%) are presented in Appendix S1: part 2.

**TABLE 2:** Number of patients diagnosed with chronic hepatitis B who were linked to care, treated and retained in care, by patient characteristics, Norway, 2008–2022.

Characteristic	All patients					Still resident in Norway at the end of 2022				
	Number of patients	Ever linked to care		Ever treated		Number of patients	Retained in care in last 12 months^a^		Treated in last 12 months^a^	
		*N*	%	*N*	%		*N*	%	*N*	%
Overall	10542	8301	79	2454	23	8979	4476	50	1528	17
**Year of diagnosis** ^b^										
2008–2009	2245	1646	73	645	29	1806	813	45	356	20
2010–2011	1517	1131	75	343	23	1233	491	40	182	15
2012–2013	1522	1156	76	322	21	1245	556	45	188	15
2014–2015	1573	1262	80	322	20	1386	660	48	212	15
2016–2017	1489	126	85	327	22	1313	655	50	221	17
2018–2019	1046	917	88	229	22	942	556	59	156	17
2020–2021	710	606	85	180	25	660	437	66	144	22
2022^c^	440	323	73	86	20	394	308	78	69	18
**Age in years** ^d^										
0–11	124	104	84	21	17	12	4	33	1	8.3
12–24	1320	1091	83	264	20	289	164	57	39	13
25–44	6052	4826	80	1331	22	4082	2133	52	587	14
45–64	2653	2013	76	722	27	3769	1827	48	731	19
≥ 65	391	266	68	116	30	827	348	42	170	21
Unknown	2	1	50	0	0	0	—	—	—	—
**Sex**										
Female	4458	3563	80	913	20	3955	2003	51	606	15
Male	6065	4726	78	1539	25	5010	2467	49	920	18
Unknown	19	12	63	2	11	14	6	43	2	14
**Region of residence** ^e^										
Mid Norway	728	586	80	172	24	589	314	53	111	19
Northern Norway	748	565	76	185	25	435	220	51	98	23
Oslo	2690	2261	84	598	22	2246	1111	49	339	15
South‐east Norway (excluding Oslo)	4142	3325	80	964	23	3980	1957	49	679	17
Western Norway	2162	1554	72	531	25	1679	845	50	294	18
Unknown	72	10	14	4	5.6	50	29	58	7	14
**Place of birth** ^f^										
Norway	1519	917	60	380	25	1126	333	30	144	13
North, West and Southern Europe (excluding Norway)	704	541	77	142	20	594	312	53	91	15
America	60	43	72	11	18	49	22	45	7	14
Central and Eastern Asia	589	487	83	207	35	509	313	61	155	30
Eastern Europe	931	709	76	152	16	779	382	49	91	12
North Africa and Western Asia	986	833	84	213	22	903	461	51	140	16
South‐eastern Asia and Oceania	2116	1791	85	738	35	1938	1154	60	543	28
Southern Asia	817	680	83	212	26	706	358	51	135	19
Sub‐Saharan Africa	2702	2217	82	385	14	2375	1141	48	222	9.3
Unknown	118	83	70	14	12	0	—	—	—	—
**Residence status at the end of 2022**										
Resident	8979	7443	83	2176	24	—	—	—	—	—
Died or out‐migrated	1373	744	54	258	19	—	—	—	—	—
Unknown	190	114	60	20	11	—	—	—	—	—

^a^ Last 12 months refers to the last 12 months of the study period, July 2022–June 2023.

^b^ For patients identified in NPR (*n* = 4816), the date of first consultation for chronic hepatitis B is used as a proxy for the date of diagnosis. The Appendix S1: part 1 shows that CHB consultation dates in NPR closely reflect diagnosis dates in MSIS. However, one important exception will be 2008–2009, as patients identified in NPR for these years will include a notable, yet unknown, proportion who were diagnosed before 2008, and for whom the first consultation for chronic hepatitis B in our dataset reflects a consultation for clinical follow‐up.

^c^ For 2022, linkage to care and treatment uptake may be underestimated, as follow‐up data were only available until June 2023.

^d^ For all patients this is the age at chronic hepatitis B diagnosis. For patients still resident at the end of 2022, this is the age as of 2023.

^e^ For all patients, this is the region of residence at chronic hepatitis B diagnosis. For patients still resident at the end of 2022, this is the current region of residence.

^f^ Categorised according to UN groupings: https://unstats.un.org/unsd/methodology/m49.

### 3.2 Linkage to Care

Among all diagnosed PLWHB, 8301 (79%) had ever been LTC (Table 2). Of the 8301, 7763 (94%) were defined as LTC by an NPR consultation date, and 539 (6.5%) by a treatment prescription date. Of the 539, 409 (76%) also had a LTC‐defining consultation in NPR after their first treatment prescription. For the remaining 130, the medicine received is presented in Appendix S1: part 3.

For 3957 MSIS patients, the median time from diagnosis to LTC decreased from 1.9 years (IQR: 0.5–6.3) in 2008–2009 to 0.2 years (IQR: 0.1–0.3) from 2018 onwards (Appendix S1: part 4).

In multivariable models, diagnosed PLWHB born outside Norway had a 24% (RR: 1.24, 95% CI: 1.19–1.29) higher probability of being LTC compared to patients born in Norway. There was also a slightly higher probability of LTC among PLWHB diagnosed in 2018–2019 (RR: 1.04, 95% CI: 1.01–1.06 compared to 2016–2017) and PLWHB aged 0–24 years (RR: 1.03, 95% CI: 1.01–1.05, compared to patients 25–44 years). Patients who were no longer resident in Norway had a 29% (RR: 0.71, 95% CI: 0.68–0.75) lower probability of being LTC, compared to residents. There was also a slightly lower probability of being LTC among PLWHB diagnosed before 2016 or resident outside Oslo, with effect sizes ranging from 4% (RR: 0.96, 95% CI: 0.93–0.99) to 14% (RR: 0.86, 95% CI: 0.84–0.89) (Table 3).

**TABLE 3:** Relative risk of being linked to care from univariable and multivariable log‐binomial regression, patients diagnosed with chronic hepatitis B, Norway, 2008–2022.

Characteristic	Linked to care^a^		Univariable			Multivariable		
	Yes	No	RR	95% CI	*p*	RR	95% CI	*p*
**Year of diagnosis** ^b^								
2016–2017	1243	224	Ref	—	—	Ref	—	—
2008–2009	1629	540	0.89	0.86–0.92	< 0.001	0.95	0.92–0.97	< 0.001
2010–2011	1114	382	0.88	0.85–0.91	< 0.001	0.93	0.90–0.96	< 0.001
2012–2013	1138	362	0.90	0.86–0.93	< 0.001	0.95	0.92–0.98	< 0.001
2014–2015	1247	308	0.95	0.92–0.98	< 0.001	0.96	0.93–0.99	0.01
2018–2019	911	121	1.04	1.01–1.07	0.01	1.04	1.01–1.06	< 0.001
2020–2021	601	96	1.02	0.98–1.06	0.35	1.00	0.97–1.04	0.85
2022^c^	319	112	0.87	0.82–0.93	< 0.001	0.89	0.84–0.94	< 0.001
**Age at diagnosis in years**								
25–44	4768	1169	Ref	—	—	Ref	—	—
0–24	1174	239	1.03	1.01–1.06	0.01	1.03	1.01–1.05	< 0.001
45–64	1996	619	0.95	0.93–0.97	< 0.001	1.00	0.98–1.02	0.82
≥ 65	264	118	0.86	0.80–0.92	< 0.001	0.95	0.89–1.00	0.07
**Sex**								
Female	3547	871	Ref	—	—	Ref	—	—
Male	4655	1274	0.98	0.96–1.00	0.03	0.99	0.98–1.01	0.48
**Region of residence at diagnosis**								
Oslo	2251	421	Ref	—	—	Ref	—	—
Mid Norway	575	140	0.95	0.92–0.99	0.02	0.94	0.91–0.97	< 0.001
Northern Norway	547	179	0.89	0.86–0.94	< 0.001	0.89	0.85–0.92	< 0.001
South‐east Norway (excluding Oslo)	3301	809	0.95	0.93–0.97	< 0.001	0.95	0.94–0.97	< 0.001
Western Norway	1527	596	0.85	0.83–0.88	< 0.001	0.86	0.84–0.89	< 0.001
**Place of birth**								
Norway	916	581	Ref	—	—	Ref	—	—
Outside Norway	7286	1564	1.35	1.29–1.40	< 0.001	1.24	1.19–1.29	< 0.001
**Residence status**								
Resident	7430	1507	Ref	—	—	Ref	—	—
Died or out‐migrated	741	597	0.67	0.63–0.70	< 0.001	0.71	0.68–0.75	< 0.001

Abbreviations: CI, confidence interval; RR, relative risk.

^a^ Linkage to care is defined as a specialist outpatient consultation or treatment for chronic hepatitis B after diagnosis.

^b^ For patients identified in NPR (*n* = 4816), the date of first consultation for chronic hepatitis B is used as a proxy for date of diagnosis. The Appendix S1: part 1 shows that CHB consultation dates in NPR closely reflect diagnosis dates in MSIS. However, one important exception will be 2008–2009, as patients identified in NPR for these years will include a notable, yet unknown, proportion who were diagnosed before 2008, and for whom the first consultation for chronic hepatitis B in our dataset reflects a consultation for clinical follow‐up.

^c^ For 2022, linkage to care may be underestimated, as follow‐up data were only available until June 2023.

Results were consistent in sensitivity analyses defining LTC including a wider range of hospital stays or relevant non‐CHB‐specific ICD‐10 codes (Appendix S1: part 5). One exception was among Norwegian‐born patients when including outpatient consultations with hepatitis C codes. In this case, LTC increased from 917 (60%) to 1090 (72%) patients (Appendix S1: part 5). However, a model defining LTC including outpatient hospital consultations with other relevant non‐CHB‐specific ICD‐10 codes (such as hepatitis C) was consistent with the main analysis, although the effect size for PLWHB born outside Norway was smaller (RR: 1.06, 95% CI: 1.03–1.09; Appendix S1: part 6). The associations observed in our other sensitivity analyses were generally consistent with the main log‐binomial model, including disaggregating patients born overseas by region of birth, restricting the model to the MSIS cohort and running a survival analysis model (Appendix S1: part 6).

### 3.3 Retention in Care

**FIGURE 1:**
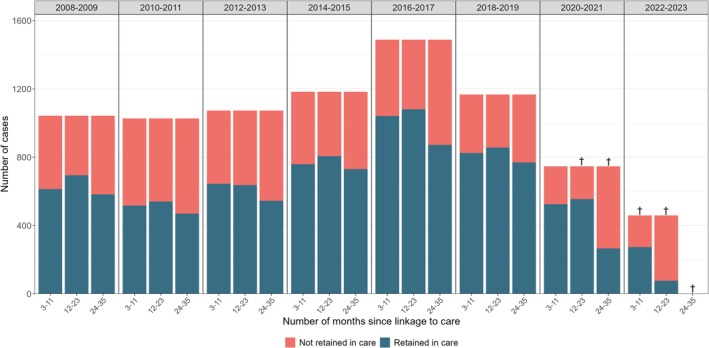
Number of patients diagnosed with chronic hepatitis B retained in care up to 35 months after hepatitis B linkage to care, by year of hepatitis B linkage to care, Norway, 2008–2022. Data on follow‐up in primary care from the Norwegian Registry for Primary Health Care prior to 2016. Therefore, retention in care will be somewhat underestimated for 2008–2015. At the end of the study period, 13% of resident patients retained in care (or 6% of all resident patients, both retained and not retained in care) had had a consultation in primary care in the last 12 months, but not a consultation in specialist care or a treatment prescription. ^†^Follow‐up is incomplete as data were available until June 2023.

Of the 8301 patients who had been LTC, 8069 (97%) were still resident in Norway 12 months after LTC. Of these, 5200 (64%) were RIC in the first 3–11 months after LTC. This proportion varied from 51% to 59% in 2008–2011, 61% to 65% in 2012–2015 and 71% to 72% in 2016–2021. For patients LTC in 2022–2023, the proportion was 60%, although follow‐up was incomplete (until June 2023). The proportion remained relatively stable in the second year (12–23 months) after LTC, varying among years with complete follow‐up (until 2018–2019) from 54% in 2010–2011 to 75% in 2016–2017 and 2018–2019. In the third year (24–35 months), the proportion among years with complete follow‐up (until 2018–2019) varied from 48% in 2010–2011 to 68% in 2018–2019 (Figure 1).

Among the diagnosed PLWHB that were registered as still resident at the end of 2022, 4476 (50%) had been RIC in the last 12 months of the study period. Of the 4476, 2913 (65% or 32% of all resident patients) had had a consultation in specialist care, 1003 (22%) had not had a consultation in specialist care but were on treatment, and 560 (13%) had had a consultation in primary care but not in specialist care and were not on treatment. Appendix S1: part 7 provides a breakdown of the data source determining RIC by diagnosis year. Results were consistent in sensitivity analyses defining RIC as the last 18 months of the study period or by including a wider range of hospital stays or relevant non‐CHB‐specific ICD‐10 codes (Appendix S1: parts 5 and 7). Among 2176 resident patients who had ever been treated, 1813 (83%) were RIC. Among 6803 resident untreated patients, 2663 (39%) were RIC.

In multivariable models, PLWHB born outside Norway had a 67% (RR: 1.67, 95% CI: 1.53–1.84) higher probability of being RIC, compared to patients born in Norway. We also found a higher probability of being RIC among PLWHB diagnosed from 2018 onwards, compared to PLWHB diagnosed in 2016–2017. There was a lower probability of being RIC among PLWHB diagnosed in 2010–2013. By age, patients ≥ 65 years had a slightly lower probability of being RIC, compared to patients 25–44 years (RR: 0.91, 95% CI: 0.84–0.99) (Table 4). The associations observed were generally consistent in a model disaggregating patients born overseas by region of birth (Appendix S1: part 6).

**TABLE 4:** Relative risk of being retained in care in the last 12 months from univariable and multivariable log‐binomial regression among patients diagnosed with chronic hepatitis B still resident at the end of 2022, Norway, 2008–2022.

Characteristic	Retained in care^a^		Univariable			Multivariable		
	Yes	No	RR	95% CI	*p*	RR	95% CI	*p*
**Year of diagnosis** ^b^								
2016–2017	652	654	Ref	—	—	Ref	—	—
2008–2009	808	988	0.90	0.84–0.97	0.006	0.95	0.88–1.02	0.14
2010–2011	487	737	0.80	0.73–0.87	< 0.001	0.83	0.76–0.90	< 0.001
2012–2013	555	684	0.90	0.83–0.97	0.010	0.91	0.84–0.98	0.020
2014–2015	656	725	0.95	0.88–1.03	0.2	0.96	0.89–1.04	0.3
2018–2019	556	386	1.18	1.10–1.28	< 0.001	1.18	1.09–1.27	< 0.001
2020–2021	435	222	1.33	1.23–1.43	< 0.001	1.32	1.22–1.42	< 0.001
2022	292	78	1.58	1.46–1.70	< 0.001	1.56	1.44–1.67	< 0.001
**Age at end of study period in years**								
25–44	2112	1932	Ref	—	—	Ref	—	—
0–24	166	133	1.06	0.95–1.18	0.3	1.02	0.92–1.12	0.7
45–64	1817	1931	0.93	0.89–0.97	< 0.001	1.00	0.96–1.05	0.9
≥ 65	346	478	0.80	0.74–0.87	< 0.001	0.91	0.84–0.99	0.030
**Sex**								
Female	1990	1944	Ref	—	—	Ref	—	—
Male	2451	2530	0.97	0.93–1.01	0.2	0.99	0.96–1.03	0.7
**Region of residence at end of study period**								
Oslo	1111	1135	Ref	—	—	Ref	—	—
Mid Norway	314	275	1.08	0.99–1.17	0.089	1.04	0.96–1.13	0.3
Northern Norway	220	214	1.02	0.92–1.13	0.6	0.95	0.87–1.05	0.4
South‐east Norway (excluding Oslo)	1952	2019	0.99	0.94–1.05	0.8	0.99	0.95–1.04	0.8
Western Norway	844	831	1.02	0.96–1.08	0.6	0.99	0.94–1.06	0.9
**Place of birth**								
Norway	333	793	Ref	—	—	Ref	—	—
Outside Norway	4108	3681	1.78	1.63–1.96	< 0.001	1.67	1.53–1.84	< 0.001

Abbreviations: CI, confidence interval; RR, relative risk.

^a^ Retention in care is defined as a specialist outpatient consultation for chronic hepatitis B, treatment for chronic hepatitis B or primary care consultation for viral hepatitis.

^b^ For patients identified in NPR (*n* = 4816), the date of first consultation for chronic hepatitis B is used as a proxy for the date of diagnosis. The Appendix S1: part 1 shows that CHB consultation dates in NPR closely reflect diagnosis dates in MSIS. However, one important exception will be 2008–2009, as patients identified in NPR for these years will include a notable, yet unknown, proportion who were diagnosed before 2008, and for whom the first consultation for chronic hepatitis B in our dataset reflects a consultation for clinical follow‐up.

### 3.4 Treatment Uptake

Among all patients, 2454 (23%) had ever received treatment, varying from 20% to 29% by diagnosis year (Table 2). Among those still resident at the end of 2022, 1528 (17%) had received treatment in the last 12 months. Tenofovir disoproxil fumarate (J05AF07) and entecavir (J05AF10) were the most common medicines prescribed. At least one of these medicines was received by 2129 (87%) of all treated patients and 1446 (95%) of all patients treated in the last 12 months. The distribution of treatment codes among treated patients is presented in Appendix S1: part 8.

## 4 Discussion

We find that 79% of patients diagnosed with CHB in Norway from 2008 to 2022 were ever LTC (i.e., specialist consultation or treatment for hepatitis B after diagnosis). The proportion increased over time from 73% in 2008–2009 to 88% in 2018–2019. Thus, over 10% of diagnosed PLWHB each year in Norway are not being seen by a specialist, contrary to the national CHB guidelines [8] and as specified in a national electronic handbook that is the preferred source for advice among general practitioners [12]. Published data on LTC are sparse on a national level. In Europe, monitoring reports from the European Centre for Disease Prevention and Control do not currently provide data on this indicator [9]. A registry‐linkage study in Denmark found that a third of PLWHB diagnosed by the end of 2016 had attended specialist care [13]. Another study in England reported that a quarter of HBsAg positive patients diagnosed from 2008 to 2019 at general practices had been referred to a specialist [14]. Globally, 95% CI from pooled estimates of LTC (defined as assessment of eligibility for treatment following a diagnosis of hepatitis B) ranged from 47% to 67% for primary or co‐managed care, 63% to 80% for specialist care and 54% to 93% for community‐based testing [15].

Following LTC, over a quarter of all diagnosed PLWHB in Norway were not RIC in the first 2 years after CHB diagnosis. In the last 12 months of the study period, 50% of resident PLWHB were still RIC. There are again sparse national data to compare with. Just three EU/EEA countries reported on the number of diagnosed PLWHB still RIC in 2022 [9]. A WHO systematic review and meta‐analysis found a similar global trend to our results, with RIC declining to 66% (95% CI: 40–85) at 48 months after diagnosis among PLWHB who had received treatment, and 42% (95% CI: 27–58) after 24 months for untreated patients [15].

Treatment uptake in our study was 23%, with 17% of resident PLWHB still on treatment at the end of the study period. Treatment uptake among diagnosed PLWHB is around 20% globally and 12% in Europe [1]. In 2022, four EU/EEA countries were able to provide data on treatment uptake among diagnosed PLWHB, ranging from 7% to 36% [9]. The reasonable treatment uptake we find may suggest that those lost to follow‐up are mainly patients with an inactive carrier state.

In 2021, the WHO estimated that about one fifth of all PLWHB would require treatment, based on treatment criteria at the time [16]. Given that treatment criteria in Norway have been more inclusive than the WHO guidelines since 2017, the proportion on treatment may be expected to be higher. While no data on treatment uptake among eligible patients are available in Norway, work is ongoing to establish a clinical hepatitis B registry at hospitals in South‐East Norway, in collaboration with a European sentinel surveillance network [17].

No country is currently on track to achieve the 2030 elimination target for hepatitis B [18] and new strategies are required. In their 2024 CHB guidelines, the WHO calls for ‘radical simplification of treatment criteria, … and care pathways’ [15]. It lays out a vision of simplified, integrated, decentralised and differentiated models of care. Our results highlight clear potential to improve the currently suboptimal clinical follow‐up of diagnosed PLWHB in Norway, a country with universal and free access to testing, specialist clinical follow‐up and treatment.

There is a need to actively identify and reengage the roughly 4500 diagnosed PLWHB we find are not currently in adequate clinical care. Routine screening of emergency admissions in New South Wales, Australia [19] and patient journal review in Barcelona, Spain [20] provide examples of such initiatives. The underlying data in this study could theoretically form a database for systematically identifying and contacting diagnosed PLHWB who have been lost to follow‐up in Norway. Consideration should be given to removing the existing legal barriers to this. Care pathways should be re‐evaluated and adapted to better ensure that diagnosed PLWHB are LTC and traced if follow‐up is interrupted. The active involvement of health professionals other than physicians [21, 22] and peer‐support workers [23] may be beneficial. Measures to engage patients in care should also be tailored to different key risk groups, the benefit of which has been demonstrated in studies in Asian immigrants in New York and New Jersey, USA [24] and in First Nations people in Australia's Northern Territory [25]. Regarding treatment eligibility, WHO recently presented simplified treatment eligibility criteria for hepatitis B, which may result in up to 50% of diagnosed PLWHB requiring treatment [15]. Experience from hepatitis C shows how simplified treatment criteria can better ensure adequate clinical follow‐up [26]. Also, point‐of‐care HBV DNA testing has been associated elsewhere with increased treatment uptake among those eligible [27] and could be considered in Norway as part of a more community‐centred approach.

Multiple factors may play a role in disengagement from care [15]. We find greater potential to improve LTC and RIC among diagnosed PLWHB who were born in Norway, a result that may reflect the fact that hepatitis B transmission among Norwegian‐born persons has been largely confined to outbreaks among people who inject drugs [3], who may be at higher risk of loss to follow‐up after diagnosis [28]. There was also a slightly higher probability of LTC among younger PLWHB and a lower probability of LTC among patients resident outside Oslo or diagnosed before 2016. At the end of the study period, resident PLWHB who were ≥ 65 years old or diagnosed in the early part of the study period were less likely to be RIC. While some of our associations are not consistent with others (e.g., the lower probability of being RIC among older patients [15, 20, 29]), it is not unexpected that factors associated with disengagement from care vary between studies and settings [30]. However, the information our study provides on why clinical follow‐up is suboptimal is limited to a few demographic characteristics. Further research is therefore needed to better ascertain what patient and systemic factors are associated with loss to follow‐up in the Norwegian setting and best inform systemic changes. Examples of other factors reported by others include lower educational status, access to medical care, models of service delivery, proximity of health services to patients place of residence, lack of physician or patient knowledge and awareness, linguistic and cultural barriers and stigma [15, 29, 31].

In this study, we have leveraged linked national registry data to study key outcomes in the clinical follow‐up of CHB in Norway. This is the first time that these registry data have been linked to study these outcomes. By using data routinely reported in national registries, the methodology provides a framework for how the outcomes can be monitored in the ongoing national surveillance of hepatitis B in Norway, something few other EU/EEA countries are currently able to report on [9]. However, there are several limitations to consider with our study.

First, we are dependent on the accuracy of coding in the registries to define both our study population and outcomes. For example, while we limited our NPR cohort to patients who had received an ICD‐10 code for CHB at least twice, we cannot validate if our entire NPR cohort contained true diagnosed PLWHB. If we also included patients with acute or resolved hepatitis B virus infection (not in need of long‐term care), we might have underestimated LTC and RIC among true PLWHB. However, a sensitivity analysis restricted to the MSIS cohort supported the main conclusions. We may also underestimate LTC and RIC if patients in care were not identified as such by our outcome definitions. For example, consultations for HIV or hepatitis C may also reflect follow‐up for hepatitis B if these codes were registered instead of a hepatitis B code and the consultation included follow‐up for both infections. Including a wider range of non‐CHB‐specific ICD‐10 codes (particularly hepatitis C codes) increased the proportion LTC among Norwegian‐born patients from 60% to 72%. However, in a model defining LTC using these codes, patients born outside Norway still had a higher probability of LTC than patients born in Norway. Other sensitivity analyses on our outcome definitions did not notably affect the results. Second, we are dependent on the availability of data. We did not have data on follow‐up in primary care prior to 2016, which may explain why we saw that patients LTC before 2016 had a higher risk of loss to follow‐up in the first 2 years after linkage, compared to PLWHB diagnosed from 2016. We also do not know exactly what follow‐up patients received at each consultation and whether guideline‐recommended care was received. Furthermore, we do not know how accurately the available primary care code D72 (non‐specific for hepatitis B) reflected consultations for hepatitis B in primary care, whether those retained in primary care only were true ‘inactive carriers’ as per national guidelines, whether treatment eligibility was assessed in accordance with guidelines, nor whether spontaneous HBsAg loss occurred that may explain why some patients are no longer in clinical follow‐up [32]. Following the recent establishment of a national microbiology database [33], data on biomarkers of hepatitis B virus infection, including hepatitis B virus DNA, HBeAg, HBsAg and hepatitis B surface antibody, may be included in subsequent data linkages and provide important nuance to our outcome definitions.

## 5 Conclusions

There is clear potential to improve the clinical follow‐up of patients diagnosed with CHB in Norway, with over 10% of diagnosed PLWHB each year still not seen by a specialist and only around half of those still resident in RIC. More research is needed to better ascertain how this follow‐up could be improved. There is a paucity of data globally on clinical follow‐up after CHB diagnosis, essential for monitoring progress towards global elimination goals. Our study provides a framework for how these outcomes can be monitored in the ongoing national surveillance of hepatitis B in Norway.

## Author Contributions

**Beatriz Valcarcel Salamanca:** conceptualisation, methodology, data curation, formal analysis, visualisation, writing – original draft, writing – review and editing. **Asgeir Johannessen:** conceptualisation, methodology, writing – review and editing. **Olav Dalgard:** conceptualisation, methodology, writing – review and editing. **Robert Whittaker:** conceptualisation, project administration, methodology, data curation, formal analysis, visualisation, writing – original draft, writing – review and editing. All co‐authors have approved the final version for submission.

## Ethics Statement

Ethical approval for this study was granted by the Regional Committees for Medical and Health Research Ethics Southeast Norway (original reference number 569083, replaced by 706180 in March 2024). The need for informed consent was waived.

## Conflicts of Interest

The authors declare no conflicts of interest.

## Supporting information


**Appendix S1:** jvh70089‐sup‐0001‐AppendixS1.docx.

## Data Availability

The dataset analysed in the study contains individual‐level linked data from national registries in Norway. Anyone is freely able to apply for access to data from the same registries for research purposes, as per normal procedure for conducting health research on registry data in Norway.
