# Supplementary material for: Linkage to Care, Retention in Care and Treatment Uptake Among Patients Diagnosed With Chronic Hepatitis B in Norway, 2008–2022
Source: J Viral Hepat. 2025 Sep 22;32(10):e70089. doi: 10.1111/jvh.70089 (PMC12452215; doi:10.1111/jvh.70089)
Supplement: Supplementary file 1 — Appendix S1: jvh70089‐sup‐0001‐AppendixS1.docx. [file JVH-32-0-s001.docx]

**Linkage to care, retention in care and treatment uptake among patients diagnosed with chronic hepatitis B in Norway, 2008 – 2022.**

**Supplement**

**Contents**

[**1.** **Difference in the number of months between MSIS and NPR diagnosis dates** 2](#_Toc201829249)

[**2.** **Descriptive tables by data source used to identify the study population** 3](#_Toc201829250)

[**3.** **Patients identified as linked to care by treatment codes only** 7](#_Toc201829251)

[**4.** **Time from diagnosis to linkage to care** 8](#_Toc201829252)

[**5.** **Sensitivity analysis of the codes defining linkage to care and retention in care** 9](#_Toc201829253)

[**6.** **Sensitivity analyses for log-binomial regression models** 11](#_Toc201829254)

[**7.** **Data source determining retention in care** **in the last 12 months of the study period** 18](#_Toc201829255)

[**8.** **Distribution of ATC codes among treated patients** 19](#_Toc201829256)

# **Difference in the number of months between MSIS and NPR diagnosis dates**

In this study, diagnosed people living with hepatitis B (PLWHB) were first identified as those who had been notified to MSIS with chronic hepatitis B (CHB). However, given low completeness of data on national identity number in MSIS in the earlier part of the study period (56 – 60% from 2008 – 2010, 63 – 69% from 2011 – 2016, 80 – 93% from 2017 – 2022), we supplemented this with all persons registered in NPR with the ICD-10 codes B18.0 or B18.1 (CHB with or without hepatitis D) and a linkable national identity number who were not in the MSIS cohort.

As MSIS is a passive surveillance system, patients diagnosed with CHB may be first notified after their initial diagnosis. For instances, patients may not be notified when first diagnosed, and then subsequently notified when tested during clinical follow-up. In such patients, the first date of B18.0 or B18.1 consultation may be before the date of first MSIS diagnosis.

In Table S1 and S2 below, the time between first diagnosis date in MSIS and first B18.0 or B18.1 consultation in NPR for patients identifiable in both data sources is presented. In Table S1, the short time between these two dates indicates that the date of B18.0 or B18.1 consultation is a reasonable proxy for first diagnosis date, especially in the latter part of the study period. In Table S2, the small total number of patients indicates that the diagnosis date in MSIS reflects the first CHB diagnosis date in Norway for most patients, particularly at the end of the study period.

*Table S1. Median number of years from hepatitis B diagnosis date in MSIS to first chronic hepatitis B consultation date in NPR (ICD-10 code B18.0 or B18.1) by year of MSIS diagnosis, among patients with a diagnosis date in MSIS in the same month as or before chronic hepatitis B consultation date in NPR.*

| **Year of MSIS diagnosis** | **N** | **%** | **Median nr. years** | **Interquartile range** |
| --- | --- | --- | --- | --- |
| 2008 – 2009 | 590 | 14.4 | 1.5 | 0.3 – 5.4 |
| 2010 – 2011 | 588 | 14.0 | 0.8 | 0.3 – 3.2 |
| 2012 – 2013 | 607 | 15.0 | 0.7 | 0.2 – 2.1 |
| 2014 – 2015 | 718 | 18.2 | 0.6 | 0.2 – 1.7 |
| 2016 – 2017 | 628 | 15.0 | 0.3 | 0.2 – 0.8 |
| 2018 – 2019 | 463 | 11.3 | 0.2 | 0.1 – 0.3 |
| 2020 – 2021 | 301 | 7.3 | 0.2 | 0.1 – 0.3 |
| 2022 | 202 | 4.9 | 0.2 | 0.1 – 0.3 |
| Overall | 4,097 | 100.0 | 0.4 | 0.2 – 1.4 |

*Table S2. Median number of years from first chronic hepatitis B consultation date in NPR (ICD – 10 code B18.0 or B18.1) to hepatitis B diagnosis date in MSIS by year of NPR consultation, among patients with a chronic hepatitis B consultation date in NPR before diagnosis date in MSIS.*

| **Year of first CHB consultation in NPR** | **N** | **%** | **Median nr. years** | **Interquartile range** |
| --- | --- | --- | --- | --- |
| 2008 – 2009 | 64 | 29.9 | 4.5 | 1.6 – 7.0 |
| 2010 – 2011 | 35 | 16.3 | 4.1 | 1.4 – 5.7 |
| 2012 – 2013 | 30 | 14.0 | 2.5 | 0.6 – 5.9 |
| 2014 – 2015 | 24 | 11.2 | 2.0 | 0.4 – 5.8 |
| 2016 – 2017 | 32 | 14.9 | 1.4 | 0.7 – 3.5 |
| 2018 – 2019 | 17 | 7.9 | 1.6 | 0.2 – 2.7 |
| 2020 – 2021 | 9 | 4.2 | 0.8 | 0.2 – 1.0 |
| 2022 | 3 | 1.7 | 0.2 | 0.1 – 0.4 |
| Overall | 214 | 100.0 | 2.4 | 0.8 – 5.3 |

# **Descriptive tables by data source used to identify the study population**

Equivalent descriptive tables to Table 2 in the manuscript by the data source used to identify the patients (MSIS or NPR) are presented below (Tables S3 – S4). In this study, patients were first identified as those who had been notified to MSIS with CHB. However, given low completeness of data on identity number in MSIS in the earlier part of the study period (56 – 60% from 2008 – 2010, 63 – 69% from 2011 – 2016, 80 – 93% from 2017 – 2022), we supplemented this with all patients in NPR with the ICD-10 codes B18.0 or B18.1 (CHB with or without hepatitis D) registered at least twice and a linkable national identity number who were not in the MSIS cohort. MSIS is also a passive surveillance system, with risk for underreporting. In Norway, all newly diagnosed patients diagnosed with CHB should be referred to specialist care and patients registered in NPR soon after initial diagnosis.

First diagnosis dates among MSIS patients generally reflect first diagnosis dates in Norway. First diagnosis dates in NPR reflect the first B18.0 or B18.1 consultation from 2008 onwards, which does not necessarily reflect the timing of first CHB diagnosis in Norway. This is most likely for NPR patients with a first B18.0 or B18.1 consultation date in the early part of the study period (see part 1). The NPR cohort therefore contains patients first diagnosed before 2008, when there were notable outbreaks of hepatitis B among people who inject drugs in Norway, see the [2024 annual surveillance report on blood and sexually transmitted infections](https://www.fhi.no/publ/2025/arsrapport-2024---blod--og-seksuelt-overforbare-infeksjoner/).

This explains why key differences between the patients identified in MSIS and NPR include that patients in NPR were more likely diagnosed earlier in the study period (28% in 2008 – 2009 vs. 16% among MSIS patients), older (39% aged ≥45 years vs. 21% among MSIS patients) and born in Norway (23% vs. 7% among MSIS patients). As patients identified in NPR all had had at least two consultations in specialist healthcare for hepatitis B, it is expected that the proportion ever linked to care or treated is higher than among MSIS patients. Results for the two outcomes among those still resident were more comparable between data sources.

*Table S3. Number of patients diagnosed with chronic hepatitis B identified in MSIS linked to care, treated and retained in care, by different case characteristics, Norway, 2008 – 2022.*

| **Characteristic** | **All patients** | | | | | **Still resident in Norway at the end of 2022** | | | | |
| --- | --- | --- | --- | --- | --- | --- | --- | --- | --- | --- |
|  | **Number of patients** | **Ever linked to care** | | **Ever treated** | | **Number of patients** | **Retained in care in last 12 months^†^** | | **Treated in last 12 months^†^** | |
|  |  | **N** | **%** | **N** | **%** |  | **N** | **%** | **N** | **%** |
| Overall | 5,726 | 4,130 | 72% | 1,105 | 19% | 4,911 | 2,478 | 50% | 750 | 15% |
| *Year of diagnosis* | | | | | | | | | | |
| 2008 – 2009 | 914 | 604 | 66% | 189 | 21% | 748 | 314 | 42% | 116 | 16% |
| 2010 – 2011 | 845 | 582 | 69% | 159 | 19% | 708 | 290 | 41% | 98 | 14% |
| 2012 – 2013 | 871 | 604 | 69% | 177 | 20% | 731 | 338 | 46% | 115 | 16% |
| 2014 – 2015 | 967 | 712 | 74% | 165 | 17% | 848 | 413 | 49% | 115 | 14% |
| 2016 – 2017 | 840 | 650 | 77% | 160 | 19% | 738 | 380 | 51% | 110 | 15% |
| 2018 – 2019 | 582 | 474 | 81% | 112 | 19% | 509 | 317 | 62% | 83 | 16% |
| 2020 – 2021 | 398 | 307 | 77% | 96 | 24% | 360 | 234 | 65% | 76 | 21% |
| 2022^‡^ | 309 | 197 | 64% | 47 | 15% | 269 | 192 | 71% | 37 | 14% |
| *Age in years*^§^ | | | | | | | | | | |
| 0 – 11 | 66 | 46 | 70% | 5 | 7.6% | 10 | 4 | 40% | 1 | 10% |
| 12 – 24 | 855 | 655 | 77% | 155 | 18% | 188 | 102 | 54% | 24 | 13% |
| 25 – 44 | 3,621 | 2,664 | 74% | 665 | 18% | 2,684 | 1,397 | 52% | 343 | 13% |
| 45 – 64 | 1,006 | 662 | 66% | 233 | 23% | 1,698 | 824 | 49% | 312 | 18% |
| ≥65 | 178 | 103 | 58% | 47 | 26% | 331 | 151 | 46% | 70 | 21% |
| *Sex* | | | | | | | | | | |
| Female | 2,410 | 1,748 | 73% | 401 | 17% | 2,122 | 1,075 | 51% | 280 | 13% |
| Male | 3,299 | 2,371 | 72% | 702 | 21% | 2,775 | 1,397 | 50% | 468 | 17% |
| Unknown | 17 | 11 | 65% | 2 | 12% | 14 | 6 | 43% | 2 | 14% |
| *Region of residence^¶^* | | | | | | | | | | |
| Mid Norway | 405 | 292 | 72% | 78 | 19% | 325 | 175 | 54% | 55 | 17% |
| Northern Norway | 519 | 354 | 68% | 110 | 21% | 283 | 137 | 48% | 56 | 20% |
| Oslo | 1,338 | 1,040 | 78% | 248 | 19% | 1,132 | 593 | 52% | 159 | 14% |
| South-east Norway (excluding Oslo) | 2,193 | 1,651 | 75% | 433 | 20% | 2,157 | 1,095 | 51% | 329 | 15% |
| Western Norway | 1,260 | 788 | 63% | 235 | 19% | 975 | 456 | 47% | 148 | 15% |
| Unknown | 11 | 5 | 45% | 1 | 9.1% | 39 | 22 | 56% | 3 | 7.7% |
| *Place of birth^††^* | | | | | | | | | | |
| Norway | 416 | 253 | 61% | 120 | 29% | 327 | 128 | 39% | 67 | 20% |
| North, West and Southern Europe (excluding Norway) | 442 | 307 | 69% | 71 | 16% | 377 | 200 | 53% | 47 | 12% |
| America | 27 | 15 | 56% | 4 | 15% | 24 | 9 | 38% | 4 | 17% |
| Central and Eastern Asia | 341 | 246 | 72% | 98 | 29% | 277 | 161 | 58% | 76 | 27% |
| Eastern Europe | 722 | 512 | 71% | 98 | 14% | 592 | 296 | 50% | 64 | 11% |
| North Africa and Western Asia | 617 | 479 | 78% | 110 | 18% | 569 | 279 | 49% | 73 | 13% |
| South-eastern Asia and Oceania | 1,053 | 783 | 74% | 292 | 28% | 946 | 523 | 55% | 218 | 23% |
| Southern Asia | 475 | 355 | 75% | 108 | 23% | 405 | 213 | 53% | 82 | 20% |
| Sub-Saharan Africa | 1,599 | 1,172 | 73% | 202 | 13% | 1,394 | 669 | 48% | 119 | 8.5% |
| Unknown | 34 | 8 | 24% | 2 | 5.9% | 0 | – | – | – | – |
| *Residence status at the end of 2022* | | | | | | | | | | |
| Resident | 4,911 | 3,740 | 76% | 1,002 | 20% | – | – | – | – | – |
| Non-resident | 709 | 351 | 50% | 95 | 13% | – | – | – | – | – |
| Unknown | 106 | 39 | 39 | 8 | 8 | – | – | – | – | – |

*^†^ Last 12 months refers to the last 12 months of the study period, July 2022 – June 2023.*

*^‡^ For 2022, linkage to care and treatment uptake may be underestimated, as follow-up data was only available until June 2023.*

*^§^ For all patients this is the age at chronic hepatitis B diagnosis. For patients still resident at the end of 2022, this is the age as of 2023.*

*^¶^ For all patients this is the region of residence at chronic hepatitis B diagnosis. For patients still resident at the end of 2022, this is the current region of residence.*

*^††^ Categorised according to UN groupings: <https://unstats.un.org/unsd/methodology/m49>.*

*Table S4. Number of patients diagnosed with chronic hepatitis B identified in NPR linked to care, treated and retained in care, by different case characteristics, Norway, 2008 – 2022.*

| **Characteristic** | **All patients** | | | | | **Still resident in Norway at the end of 2022** | | | | |
| --- | --- | --- | --- | --- | --- | --- | --- | --- | --- | --- |
|  | **Number of patients** | **Ever linked to care** | | **Ever treated** | | **Number of patients** | **Retained in care in last 12 months^†^** | | **Treated in last 12 months^†^** | |
|  |  | **N** | **%** | **N** | **N** |  | **N** | **N** | **N** | **%** |
| Overall | 4,816 | 4,171 | 87% | 1,349 | 28% | 4,068 | 1,998 | 49% | 778 | 19% |
| *Year of diagnosis*^‡^ | | | | | | | | | | |
| 2008 – 2009 | 1,331 | 1,042 | 78% | 456 | 34% | 1,058 | 499 | 47% | 240 | 23% |
| 2010 – 2011 | 672 | 549 | 82% | 184 | 27% | 525 | 201 | 38% | 84 | 16% |
| 2012 – 2013 | 651 | 552 | 85% | 145 | 22% | 514 | 218 | 42% | 73 | 14% |
| 2014 – 2015 | 606 | 550 | 91% | 157 | 26% | 538 | 247 | 46% | 97 | 18% |
| 2016 – 2017 | 649 | 610 | 94% | 167 | 26% | 575 | 275 | 48% | 111 | 19% |
| 2018 – 2019 | 464 | 443 | 95% | 117 | 25% | 433 | 239 | 55% | 73 | 17% |
| 2020 – 2021 | 312 | 299 | 96% | 84 | 27% | 300 | 203 | 68% | 68 | 23% |
| 2022^§^ | 131 | 126 | 96% | 39 | 30% | 125 | 116 | 93% | 32 | 26% |
| *Age in years^¶^* | | | | | | | | | | |
| 0 – 11 | 58 | 58 | 100% | 16 | 28% | 2 | 0 | 0% | 0 | 0% |
| 12 – 24 | 465 | 436 | 94% | 109 | 23% | 101 | 62 | 61% | 15 | 15% |
| 25 – 44 | 2,431 | 2,162 | 89% | 666 | 27% | 1398 | 736 | 53% | 244 | 17% |
| 45 – 64 | 1,647 | 1,351 | 82% | 489 | 30% | 2071 | 1,003 | 48% | 419 | 20% |
| ≥65 | 213 | 163 | 77% | 69 | 32% | 496 | 197 | 40% | 100 | 20% |
| Unknown | 2 | 1 | 50% | 0 | 0% | 0 | – | – | – | – |
| *Sex* | | | | | | | | | | |
| Female | 2,048 | 1,815 | 89% | 512 | 25% | 1,833 | 928 | 51% | 326 | 18% |
| Male | 2,766 | 2,355 | 85% | 837 | 30% | 2,235 | 1,070 | 48% | 452 | 20% |
| Unknown | 2 | 1 | 50% | 0 | 0% | 0 | – | – | – | – |
| *Region of residence^††^* | | | | | | | | | | |
| Mid Norway | 323 | 294 | 91% | 94 | 29% | 264 | 139 | 53% | 56 | 21% |
| Northern Norway | 229 | 211 | 92% | 75 | 33% | 152 | 83 | 55% | 42 | 28% |
| Oslo | 1,352 | 1,221 | 90% | 350 | 26% | 1,114 | 518 | 46% | 180 | 16% |
| South-east Norway (excluding Oslo) | 1,949 | 1,674 | 86% | 531 | 27% | 1,823 | 862 | 47% | 350 | 19% |
| Western Norway | 902 | 766 | 85% | 296 | 33% | 704 | 389 | 55% | 146 | 21% |
| Unknown | 61 | 5 | 8.2% | 3 | 4.9% | 11 | 7 | 64% | 4 | 36% |
| *Place of birth^‡‡^* | | | | | | | | | | |
| Norway | 1,103 | 664 | 60% | 260 | 24% | 799 | 205 | 26% | 77 | 9.6% |
| North, West and Southern Europe (excluding Norway) | 262 | 234 | 89% | 71 | 27% | 217 | 112 | 52% | 44 | 20% |
| America | 33 | 28 | 85% | 7 | 21% | 25 | 13 | 52% | 3 | 12% |
| Central and Eastern Asia | 248 | 241 | 97% | 109 | 44% | 232 | 152 | 66% | 79 | 34% |
| Eastern Europe | 209 | 197 | 94% | 54 | 26% | 187 | 86 | 46% | 27 | 14% |
| North Africa and Western Asia | 369 | 354 | 96% | 103 | 28% | 334 | 182 | 54% | 67 | 20% |
| South-eastern Asia and Oceania | 1,063 | 1,008 | 95% | 446 | 42% | 992 | 631 | 64% | 325 | 33% |
| Southern Asia | 342 | 325 | 95% | 104 | 30% | 301 | 145 | 48% | 53 | 18% |
| Sub-Saharan Africa | 1,103 | 1,045 | 95% | 183 | 17% | 981 | 472 | 48% | 103 | 10% |
| Unknown | 84 | 75 | 89% | 12 | 14% | 0 | – | – | – | – |
| *Residence status at the end of 2022* | | | | | | | | | | |
| Resident | 4,068 | 3,703 | 91% | 1,174 | 29% | – | – | – | – | – |
| Non-resident | 664 | 393 | 59% | 163 | 25% | – | – | – | – | – |
| Unknown | 84 | 75 | 75 | 12 | 12 | – | – | – | – | – |

*^†^ Last 12 months refers to the last 12 months of the study period, July 2022 – June 2023.*

*^‡^ For patients identified in NPR (n=4,816), the date of first consultation for chronic hepatitis B is used as a proxy for date of diagnosis. The supplement, part 1, shows that CHB consultation dates in NPR closely reflect diagnosis dates in MSIS. However, one important exception will be 2008 – 2009, as patients identified in NPR for these years will include a notable, yet unknown, proportion who were diagnosed before 2008, and for whom the first consultation for chronic hepatitis B in our dataset reflects a consultation for clinical follow-up.*

*^§^ For 2022, linkage to care and treatment uptake may be underestimated, as follow-up data was only available until June 2023.*

*^¶^ For all patients this is the age at chronic hepatitis B diagnosis. For patients still resident at the end of 2022, this is the age as of 2023.*

*^††^ For all patients this is the region of residence at chronic hepatitis B diagnosis. For patients still resident at the end of 2022, this is the current region of residence.*

*^‡‡^ Categorised according to UN groupings: <https://unstats.un.org/unsd/methodology/m49>.*

# **Patients identified as linked to care by treatment codes only**

*Table S6. Medicine received and year of treatment among patients defined as linked to care, based on treatment data only, i.e. they never had a linkage to care defining consultation in NPR.*

|  | **ATC code** | | | | | |
| --- | --- | --- | --- | --- | --- | --- |
| **Year of treatment** | **J05AF05** | **J05AF07** | **J05AF10** | **J05AF13** | **L03AB10** | **L03AB11** |
| 2008 – 2009 | 9 | 1 | 4 | 0 | 12 | 10 |
| 2010 – 2011 | 4 | 0 | 1 | 0 | 3 | 7 |
| 2012 – 2013 | 2 | 1 | 3 | 0 | 4 | 10 |
| 2014 – 2015 | 0 | 0 | 4 | 0 | 2 | 2 |
| 2016 – 2017 | 0 | 3 | 9 | 0 | 0 | 5 |
| 2018 – 2019 | 1 | 6 | 7 | 1 | 0 | 0 |
| 2020 – 2021 | 2 | 1 | 4 | 0 | 0 | 0 |
| 2022 – 2023^†^ | 1 | 0 | 10 | 1 | 0 | 0 |
| Overall | 19 | 12 | 42 | 2 | 21 | 34 |

*^†^ Data available until June 2023.*

# **Time from diagnosis to linkage to care**

In this study, linkage to care was defined as the first-time registration of the ICD-10 codes for CHB (B18.0 or B18.1) for an outpatient hospital consultation in medical (code 30 – 39) or paediatric (code 44) departments in NPR, or the first-time registration of one of the following ATC codes in the Norwegian Prescribed Drug Registry: antiviral treatment (J05AF05, J05AF07, J05AF08, J05AF10, J05AF11 and J05AF13) or peg-interferon alpha 2a/2b (L03AB10 and L03AB11).

For MSIS patients, we present the time from MSIS diagnosis date to linkage to care in Tables S7 and S8. We could not analyse time to linkage to care for the NPR cohort, as we did not have data on the month of diagnosis for these patients. Table S8 reflects persons who had a diagnosis date in MSIS after their first linkage to care. MSIS is a passive surveillance system, so it is not unexpected that some diagnosis dates in MSIS may reflect a positive test as part of clinical follow-up, not the actual date of first positive test in Norway if the case was not notified when initially diagnosed. The low number of total patients in Table S8, as in S2, indicates that the diagnosis date in MSIS reflects the first CHB diagnosis date in Norway for most patients, particularly at the end of the study period.

*Table S7. Median number of years from hepatitis B diagnosis date in MSIS to linkage to care by year of MSIS diagnosis, among patients with a diagnosis date in MSIS in the same month as or before linkage to care.*

| **Year of MSIS diagnosis** | **N** | **%** | **Median nr. years** | **Interquartile range** |
| --- | --- | --- | --- | --- |
| 2008 – 2009 | 552 | 13.9 | 1.9 | 0.5 – 6.3 |
| 2010 – 2011 | 555 | 14.0 | 0.8 | 0.3 – 3.6 |
| 2012 – 2013 | 581 | 14.7 | 0.7 | 0.3 – 2.4 |
| 2014 – 2015 | 693 | 17.5 | 0.6 | 0.2 – 1.7 |
| 2016 – 2017 | 622 | 15.7 | 0.3 | 0.2 – 0.9 |
| 2018 – 2019 | 462 | 11.7 | 0.2 | 0.1 – 0.3 |
| 2020 – 2021 | 301 | 7.6 | 0.2 | 0.1 – 0.3 |
| 2022 | 191 | 4.8 | 0.2 | 0.1 – 0.3 |
| Overall | 3,957 | 100.0 | 0.4 | 0.2 – 1.6 |

*Table S8. Median number of years from linkage to care to hepatitis B diagnosis date in MSIS by year of linkage to care, among patients with a linkage to care before diagnosis date in MSIS.*

| **Year of linkage to care consultation in NPR** | **N** | **%** | **Median nr. years** | **Interquartile range** |
| --- | --- | --- | --- | --- |
| 2008 – 2009 | 46 | 26.6 | 3.8 | 2.9 – 5.6 |
| 2010 – 2011 | 23 | 13.3 | 2.8 | 1.2 – 5.5 |
| 2012 – 2013 | 22 | 12.7 | 3.6 | 1.9 – 6.0 |
| 2014 – 2015 | 23 | 13.3 | 2.2 | 0.4 – 5.9 |
| 2016 – 2017 | 31 | 17.9 | 1.3 | 0.6 – 3.7 |
| 2018 – 2019 | 12 | 6.9 | 2.2 | 0.7 – 3.7 |
| 2020 – 2021 | 9 | 5.2 | 0.8 | 0.7 – 2.2 |
| 2022 | 7 | 4.0 | 0.3 | 0.1 – 0.7 |
| Overall | 173 | 100.0 | 2.8 | 0.8 – 5.1 |

# **Sensitivity analysis of the codes defining linkage to care and retention in care**

Our data sources were national registries, and outcomes therefore subject to coding practices and the accuracy of registration. Therefore, for linkage to care and retention in care we also explored how including relevant non-CHB-specific ICD-10 codes or a wider range of hospital stays in NPR impacted our results. These are presented in Table S5.

Results were consistent with the outcome definition included in the manuscript. One exception was linkage to care among Norwegian-born cases when including hepatitis C codes for outpatient consultations (1,090/1,519, 72%, compared to 60% using manuscript definition). This notable increase was not seen for other groups of codes, e.g. adding HIV codes increased linkage to care among Norwegian-born cases to 62% (939/1,519). This may indicate that a higher proportion of cases born in Norway were linked to care than our main definition suggested, if we assume that an outpatient consultation for hepatitis C also included follow-up for hepatitis B. To explore how defining our outcome using a wider range of codes impacted our results, in part 6 we present sensitivity analyses for log-binomial regression models, including outpatient hospital consultations with other hepatitis B codes, unspecific viral hepatitis codes, HIV codes and hepatitis C codes in the outcome definition.

*Table S5. Number of patients diagnosed with chronic hepatitis B linked to care and resident patients retained in care in different sensitivity analyses using different outcome definitions.*

| **Sensitivity analysis** | **All patients** | | | **Still resident** | | |
| --- | --- | --- | --- | --- | --- | --- |
|  | **Number of patients** | **Ever linked to care** | | **Number of patients** | **Retained in care in last 12 months** | |
|  |  | **N** | **%** |  | **N** | **%** |
| Outcome definition used in manuscript | 10,542 | 8,301 | 79% | 8,979 | 4,476 | 50% |
| Including outpatient hospital consultations with other hepatitis B codes^†^ and unspecific viral hepatitis codes^‡^ | 10,542 | 8,348 | 79% | 8,979 | 4,518 | 50% |
| Including outpatient hospital consultations with HIV codes^§^ | 10,542 | 8,368 | 79% | 8,979 | 4,573 | 51% |
| Including outpatient hospital consultations with hepatitis C codes^¶^ | 10,542 | 8,514 | 81% | 8,979 | 4,504 | 50% |
| Including outpatient hospital consultations with other hepatitis B codes^†^, unspecific viral hepatitis codes^‡^ and HIV codes^§^ | 10,542 | 8,415 | 80% | 8,979 | 4,615 | 51% |
| Including outpatient hospital consultations with other hepatitis B codes^†^, unspecific viral hepatitis codes^‡^, HIV codes^§^ and hepatitis C codes^¶^ | 10,542 | 8,606 | 82% | 8,979 | 4,641 | 52% |
| Including outpatient and day-stay inpatient consultations | 10,542 | 8,383 | 80% | 8,979 | 4,476 | 50% |
| Including outpatient and day-stay inpatient hospital consultations with other hepatitis B codes^†^ and unspecific viral hepatitis codes^‡^ | 10,542 | 8,435 | 80% | 8,979 | 4,519 | 50% |
| Including outpatient and day-stay inpatient hospital consultations with other hepatitis B codes^†^, unspecific viral hepatitis codes^‡^, HIV codes^§^ and hepatitis C codes^¶^ | 10,542 | 8,686 | 82% | 8,979 | 4,641 | 52% |

*^†^ Other hepatitis B codes: ICD-10 codes B16, B17.0 and B19.1. We tested these codes, as they may have been registered instead of B18.0 or B18.1 by mistake.*

*^‡^ Other unspecific viral hepatitis codes: ICD-10 codes B17.9, B18.9 and B19. We tested these codes, as they may have been registered instead of B18.0 or B18.1 by mistake.*

*^§^ HIV codes: ICD-10 codes B20 – B24 and Z21. We tested HIV codes as patients co-infected with HIV and hepatitis B may have an HIV code registered ahead of a hepatitis B code, if a consultation included follow-up for both infections.*

*^¶^ Hepatitis C codes: ICD-10 codes B17.1, B18.2, B19.2. We tested hepatitis C codes as patients co-infected with hepatitis C and hepatitis B may have a hepatitis C code registered ahead of a hepatitis B code, if a consultation included follow-up for both infections. The code for chronic hepatitis C (B18.2) is also very similar to the codes for chronic hepatitis B (B18.0/B18.1), and may have been registered by mistake.*

# **Sensitivity analyses for log-binomial regression models**

We conducted several sensitivity analyses for linkage to care to explore how other models affected our results. As linkage to care among Norwegian-born persons was higher when a wider range of relevant non-CHB-specific ICD-10 codes were included, particularly hepatitis C codes (see part 5), we also ran a model defining linkage to care including other hepatitis B codes, unspecific viral hepatitis codes, HIV codes and hepatitis C codes (Table S9). We also ran a model disaggregating patients born overseas by region of birth (Table S10). Furthermore, we ran a model only including the MSIS cohort, as we could not validate that our entire NPR cohort contained true CHB patients and that the date of first B18.0/B18.1 consultation reflected the real date of first diagnosis. For the MSIS cohort, we ran both log-binomial regression (as per the main analysis, Table S11) and a sub-distribution hazard model (Table S12), as we had data on time from diagnosis to linkage to care for MSIS patients (see the supplement, part 4). The sub-distribution hazard model estimates sub-distribution hazard ratios (SHR), which can be interpreted similarly to hazard ratios derived by Cox proportional models, but take into consideration the hazard of competing events, such as out-migration on death (i.e. the variable ‘residence status’). The software packages survival/cmprsk in R were used to perform the sub-distribution hazard model.

The associations observed in all our sensitivity analyses were generally consistent with the main analysis. Redefining linkage to care using a wider range of codes gave a lower difference in the probability of being linked to care among patients born outside Norway, compared to Norwegian-born patients (wider range of codes RR: 1.06, 95% CI: 1.03 – 1.09; main analysis RR: 1.24, 95% CI: 1.19 – 1.29). The RR among patients ≥65 years was also statistically significant (p value 0.04 vs. 0.07 in main analysis), but unchanged (main analysis RR: 0.95, 95% CI: 0.89 – 1.00). By region of birth, all regions had a higher probability of being linked to care than patients born in Norway, except America, a cohort of only 60 patients. Restricting the cohort to MSIS patients gave a smaller difference in the risk of not being linked to care among patients born abroad, compared to Norwegian-born patients (MSIS cohort only RR: 1.10, 95% CI: 1.02 – 1.19; main analysis RR: 1.24, 95% CI: 1.19 – 1.29). There was also a lower probability of being linked to care among MSIS patients aged 45 – 64 years and ≥65 years, compared to patients aged 25 – 44 years. In the sub-distribution hazard model, there was no significant difference in the hazard ratio between patients aged 0 – 24 years and patients aged 25 – 44 years, although estimates were similar to the main model. Otherwise, results were similar to the log-binomial regression using MSIS patients.

For retention in care in the last 12 months of the study period, we ran a model disaggregating patients born overseas by region of birth (Table S13). Patients diagnosed in 2008 – 2009 had a slightly lower probability of being retained in care (RR: 0.92, 95% CI: 0.95 – 0.99), compared to patients diagnosed in 2016 – 2017, and men had a slightly higher probability of being retained in care than women (RR: 1.05, 95% CI: 1.01 – 1.09). Estimates were similar in the main model, but statistically insignificant. Otherwise, associations were consistent with the main model.

*Table S9. Relative risk of being linked to care from univariable and multivariable log-binomial regression, patients diagnosed with chronic hepatitis B, Norway, 2008 – 2022, linkage to care defined using a wider range of codes including outpatient hospital consultations with other hepatitis B codes, unspecific viral hepatitis codes, HIV codes and hepatitis C codes.*

| **Characteristic** | **Linked to care^†^** | | **Univariable** | | | **Multivariable** | | |
| --- | --- | --- | --- | --- | --- | --- | --- | --- |
|  | **Yes** | **No** | **RR** | **95% CI** | **Yes** | **No** | **RR** | **P value** |
| **Year of diagnosis**^‡^ | | | | | | | | |
| 2016 – 2017 | 1,266 | 201 | Ref | — | — | Ref | — | — |
| 2008 – 2009 | 1,720 | 449 | 0.92 | 0.89 – 0.95 | <0.001 | 0.95 | 0.92 – 0.97 | <0.001 |
| 2010 – 2011 | 1,164 | 332 | 0.90 | 0.87 – 0.93 | <0.001 | 0.93 | 0.90 – 0.96 | <0.001 |
| 2012 – 2013 | 1,192 | 308 | 0.92 | 0.89 – 0.95 | <0.001 | 0.96 | 0.93 – 0.98 | 0.002 |
| 2014 – 2015 | 1,276 | 279 | 0.95 | 0.92 – 0.98 | 0.001 | 0.96 | 0.93 – 0.98 | 0.002 |
| 2018 – 2019 | 936 | 96 | 1.05 | 1.02 – 1.08 | <0.001 | 1.04 | 1.02 – 1.06 | <0.001 |
| 2020 – 2021 | 615 | 82 | 1.02 | 0.99 – 1.06 | 0.20 | 1.01 | 0.98 – 1.04 | 0.56 |
| 2022^§^ | 335 | 96 | 0.90 | 0.85 – 0.95 | <0.001 | 0.91 | 0.86 – 0.96 | <0.001 |
| **Age at diagnosis in years** | | | | | | | | |
| 25 – 44 | 4,906 | 1,031 | Ref | — | — | Ref | — | — |
| 0 – 24 | 1,196 | 217 | 1.02 | 1.00 – 1.05 | 0.06 | 1.02 | 1.00 – 1.04 | 0.02 |
| 45 – 64 | 2,122 | 493 | 0.98 | 0.96 – 1.00 | 0.10 | 1.00 | 0.98 – 1.02 | 0.78 |
| ≥65 | 280 | 102 | 0.89 | 0.83 – 0.94 | <0.001 | 0.94 | 0.89 – 1.00 | 0.04 |
| **Sex** | | | | | | | | |
| Female | 3,628 | 790 | Ref | — | — | Ref | — | — |
| Male | 4,876 | 1,053 | 1.00 | 0.98 – 1.02 | 0.87 | 1.00 | 0.99 – 1.02 | 0.90 |
| **Region of residence at diagnosis** | | | | | | | | |
| Oslo | 2,322 | 350 | Ref | — | — | Ref | — | — |
| Mid Norway | 589 | 126 | 0.95 | 0.91 – 0.98 | 0.005 | 0.94 | 0.91 – 0.97 | <0.001 |
| Northern Norway | 570 | 157 | 0.90 | 0.87 – 0.94 | <0.001 | 0.90 | 0.87 – 0.93 | <0.001 |
| South-east Norway (excluding Oslo) | 3,432 | 678 | 0.96 | 0.94 – 0.98 | <0.001 | 0.96 | 0.94 – 0.97 | <0.001 |
| Western Norway | 1,591 | 532 | 0.86 | 0.84 – 0.89 | <0.001 | 0.87 | 0.85 – 0.89 | <0.001 |
| **Place of birth** | | | | | | | | |
| Norway | 1,107 | 390 | Ref | — | — | Ref | — | — |
| Outside Norway | 7,397 | 1,453 | 1.13 | 1.10 – 1.17 | <0.001 | 1.06 | 1.03 – 1.09 | <0.001 |
| **Residence status** | | | | | | | | |
| Resident | 7,645 | 1,292 | Ref | — | — | Ref | — | — |
| Died or out-migrated | 826 | 512 | 0.72 | 0.69 – 0.75 | <0.001 | 0.75 | 0.71 – 0.78 | <0.001 |

*CI: Confidence interval. RR: Relative risk.*

*^†^ Linkage to care is defined as a specialist outpatient consultation for chronic hepatitis B, treatment for chronic hepatitis B or an outpatient consultation for hepatitis B, unspecific viral hepatitis, HIV or hepatitis C after diagnosis.*

*^‡^ For patients identified in NPR (n=4,816), the date of first consultation for chronic hepatitis B is used as a proxy for date of diagnosis. The supplement, part 1, shows that CHB consultation dates in NPR closely reflect diagnosis dates in MSIS. However, one important exception will be 2008 – 2009, as patients identified in NPR for these years will include a notable, yet unknown, proportion who were diagnosed before 2008, and for whom the first consultation for chronic hepatitis B in our dataset reflects a consultation for clinical follow-up.*

*^§^ For 2022, linkage to care may be underestimated, as follow-up data was only available until June 2023.*

*Table S10. Relative risk of being linked to care from univariable and multivariable log-binomial regression, patients diagnosed with chronic hepatitis B, Norway, 2008 – 2022, place of birth by region.*

| **Characteristic** | **Linked to care^†^** | | **Univariable** | | | **Multivariable** | | |
| --- | --- | --- | --- | --- | --- | --- | --- | --- |
|  | **Yes** | **No** | **RR** | **95% CI** | **Yes** | **No** | **RR** | **P value** |
| **Year of diagnosis**^‡^ | | | | | | | | |
| 2016 – 2017 | 1,243 | 224 | Ref | — | — | Ref | — | — |
| 2008 – 2009 | 1,629 | 540 | 0.89 | 0.86 – 0.92 | <0.001 | 0.94 | 0.92 – 0.97 | <0.001 |
| 2010 – 2011 | 1,114 | 382 | 0.88 | 0.85 – 0.91 | <0.001 | 0.93 | 0.90 – 0.96 | <0.001 |
| 2012 – 2013 | 1,138 | 362 | 0.90 | 0.86 – 0.93 | <0.001 | 0.94 | 0.91 – 0.97 | <0.001 |
| 2014 – 2015 | 1,247 | 308 | 0.95 | 0.92 – 0.98 | <0.001 | 0.96 | 0.93 – 0.99 | 0.01 |
| 2018 – 2019 | 911 | 121 | 1.04 | 1.01 – 1.07 | 0.01 | 1.03 | 1.01 – 1.06 | 0.02 |
| 2020 – 2021 | 601 | 96 | 1.02 | 0.98 – 1.06 | 0.35 | 1.01 | 0.98 – 1.05 | 0.55 |
| 2022^§^ | 319 | 112 | 0.87 | 0.82 – 0.93 | <0.001 | 0.91 | 0.86 – 0.96 | <0.001 |
| **Age at diagnosis in years** | | | | | | | | |
| 25 – 44 | 4,768 | 1,169 | Ref | — | — | Ref | — | — |
| 0 – 24 | 1,174 | 239 | 1.03 | 1.01 – 1.06 | 0.01 | 1.03 | 1.01 – 1.05 | 0.01 |
| 45 – 64 | 1,996 | 619 | 0.95 | 0.93 – 0.97 | <0.001 | 1.00 | 0.98 – 1.02 | 0.97 |
| ≥65 | 264 | 118 | 0.86 | 0.80 – 0.92 | <0.001 | 0.96 | 0.90 – 1.01 | 0.14 |
| **Sex** | | | | | | | | |
| Female | 3,547 | 871 | Ref | — | — | Ref | — | — |
| Male | 4,655 | 1,274 | 0.98 | 0.96 – 1.00 | 0.03 | 1.00 | 0.99 – 1.02 | 0.58 |
| **Region of residence at diagnosis** | | | | | | | | |
| Oslo | 2,251 | 421 | Ref | — | — | Ref | — | — |
| Mid Norway | 575 | 140 | 0.95 | 0.92 – 0.99 | 0.02 | 0.95 | 0.91 – 0.98 | <0.001 |
| Northern Norway | 548 | 179 | 0.89 | 0.86 – 0.94 | <0.001 | 0.90 | 0.86 – 0.94 | <0.001 |
| South-east Norway (excluding Oslo) | 3,301 | 809 | 0.95 | 0.93 – 0.97 | <0.001 | 0.96 | 0.94 – 0.97 | <0.001 |
| Western Norway | 1,527 | 596 | 0.85 | 0.83 – 0.88 | <0.001 | 0.88 | 0.86 – 0.90 | <0.001 |
| **Place of birth**^¶^ | | | | | | | | |
| Norway | 916 | 581 | Ref | — | — | Ref | — | — |
| North, West and Southern Europe (excluding Norway) | 540 | 159 | 1.26 | 1.19 – 1.34 | <0.001 | 1.15 | 1.09 – 1.21 | <0.001 |
| America | 43 | 17 | 1.17 | 0.99 – 1-38 | 0.06 | 1.08 | 0.93 – 1.26 | 0.31 |
| Central and Eastern Asia | 486 | 99 | 1.36 | 1.29 – 1.43 | <0.001 | 1.23 | 1.17 – 1.29 | <0.001 |
| Eastern Europe | 709 | 219 | 1.25 | 1.18 – 1.32 | <0.001 | 1.17 | 1.11 – 1.23 | <0.001 |
| North Africa and Western Asia | 833 | 151 | 1.38 | 1.32 – 1.45 | <0.001 | 1.21 | 1.16 – 1.27 | <0.001 |
| South-eastern Asia and Oceania | 1,785 | 316 | 1.39 | 1.33 – 1.45 | <0.001 | 1.24 | 1.19 – 1.29 | <0.001 |
| Southern Asia | 680 | 134 | 1.37 | 1.30 – 1.44 | <0.001 | 1.21 | 1.16 – 1.27 | <0.001 |
| Sub-Saharan Africa | 2,210 | 469 | 1.35 | 1.29 – 1.41 | <0.001 | 1.20 | 1.16 – 1.25 | <0.001 |
| **Residence status** | | | | | | | | |
| Resident | 7,430 | 1,507 | Ref | — | — | Ref | — | — |
| Died or out-migrated | 741 | 597 | 0.67 | 0.63 – 0.70 | <0.001 | 0.75 | 0.72 – 0.79 | <0.001 |

*CI: Confidence interval. RR: Relative risk.*

*^†^ Linkage to care is defined as a specialist outpatient consultation or treatment for chronic hepatitis B after diagnosis.*

*^‡^ For patients identified in NPR (n=4,816), the date of first consultation for chronic hepatitis B is used as a proxy for date of diagnosis. The supplement, part 1, shows that CHB consultation dates in NPR closely reflect diagnosis dates in MSIS. However, one important exception will be 2008 – 2009, as patients identified in NPR for these years will include a notable, yet unknown, proportion who were diagnosed before 2008, and for whom the first consultation for chronic hepatitis B in our dataset reflects a consultation for clinical follow-up.*

*^§^ For 2022, linkage to care may be underestimated, as follow-up data was only available until June 2023.*

*^¶^ Categorised according to UN groupings: <https://unstats.un.org/unsd/methodology/m49>.*

*Table S11. Relative risk of being linked to care from univariable and multivariable log-binomial regression, patients diagnosed with chronic hepatitis B, Norway, 2008 – 2022, MSIS cohort only.*

| **Characteristic** | **Linked to care^†^** | | **Univariable** | | | **Multivariable** | | |
| --- | --- | --- | --- | --- | --- | --- | --- | --- |
|  | **Yes** | **No** | **RR** | **95% CI** | **P value** | **RR** | **95% CI** | **P value** |
| **Year of diagnosis** | | | | | | | | |
| 2016 – 2017 | 647 | 186 | Ref | — | — | Ref | — | — |
| 2008 – 2009 | 599 | 309 | 0.85 | 0.80 – 0.90 | <0.001 | 0.88 | 0.83 – 0.93 | <0.001 |
| 2010 – 2011 | 578 | 259 | 0.89 | 0.84 – 0.94 | <0.001 | 0.92 | 0.87 – 0.97 | <0.001 |
| 2012 – 2013 | 602 | 264 | 0.89 | 0.85 – 0.95 | <0.001 | 0.94 | 0.89 – 0.99 | 0.01 |
| 2014 – 2015 | 711 | 253 | 0.95 | 0.90 – 1.00 | 0.05 | 0.96 | 0.91 – 1.00 | 0.07 |
| 2018 – 2019 | 472 | 100 | 1.06 | 1.01 – 1.12 | 0.02 | 1.08 | 1.03 – 1.12 | <0.001 |
| 2020 – 2021 | 305 | 84 | 1.01 | 0.95 – 1.08 | 0.77 | 1.00 | 0.95 – 1.07 | 0.89 |
| 2022^‡^ | 195 | 107 | 0.83 | 0.76 – 0.91 | <0.001 | 0.86 | 0.79 – 0.94 | <0.001 |
| **Age at diagnosis in years** | | | | | | | | |
| 25 – 44 | 2,647 | 932 | Ref | — | — | Ref | — | — |
| 0 – 24 | 699 | 216 | 1.03 | 0.99 – 1.08 | 0.12 | 1.04 | 1.00 – 1.07 | 0.04 |
| 45 – 64 | 657 | 338 | 0.89 | 0.85 – 0.94 | <0.001 | 0.92 | 0.88 – 0.96 | <0.001 |
| ≥65 | 103 | 74 | 0.79 | 0.69 – 0.89 | <0.001 | 0.86 | 0.77 – 0.97 | 0.01 |
| **Sex** | | | | | | | | |
| Female | 1,746 | 656 | Ref | — | — | Ref | — | — |
| Male | 2,363 | 906 | 0.99 | 0.96 – 1.03 | 0.74 | 1.00 | 0.97 – 1.02 | 0.77 |
| **Region of residence at diagnosis** | | | | | | | | |
| Oslo | 1.037 | 291 | Ref | — | — | Ref | — | — |
| Mid Norway | 291 | 113 | 0.92 | 0.86 – 0.99 | 0.02 | 0.91 | 0.86 – 0.97 | <0.001 |
| Northern Norway | 353 | 162 | 0.88 | 0.82 – 0.94 | <0.001 | 0.87 | 0.82 – 0.92 | <0.001 |
| South-east Norway (excluding Oslo) | 1.642 | 535 | 0.97 | 0.93 – 1.00 | 0.07 | 0.95 | 0.92 – 0.98 | <0.001 |
| Western Norway | 786 | 461 | 0.81 | 0.77 – 0.85 | <0.001 | 0.81 | 0.77 – 0.85 | <0.001 |
| **Place of birth** | | | | | | | | |
| Norway | 253 | 163 | Ref | — | — | Ref | — | — |
| Outside Norway | 3,856 | 1,399 | 1.21 | 1.12 – 1.31 | 0.00 | 1.10 | 1.02 – 1.19 | 0.01 |
| **Residence status** | | | | | | | | |
| Resident | 3,728 | 1,166 | Ref | — | — | Ref | — | — |
| Died or out-migrated | 350 | 355 | 0.65 | 0.60 – 0.70 | <0.001 | 0.68 | 0.63 – 0.73 | <0.001 |

*CI: Confidence interval. RR: Relative risk.*

*^†^ Linkage to care is defined as a specialist outpatient consultation or treatment for chronic hepatitis B after diagnosis.*

*^‡^ For 2022, linkage to care may be underestimated, as follow-up data was only available until June 2023.*

*Table S12. Hazard ratio for not being linked to care from univariable and multivariable sub-distribution hazard model, patients diagnosed with chronic hepatitis B, Norway, 2008 – 2022, MSIS cohort only.*

| **Characteristic** | **Linked to care^†^** | | **Died or outmigrated before linkage to care** | **Univariable** | | | **Multivariable** | | |
| --- | --- | --- | --- | --- | --- | --- | --- | --- | --- |
|  | **Yes** | **No** |  | **SHR** | **95% CI** | **P value** | **SHR** | **95% CI** | **P value** |
| **Year of diagnosis** | | | | | | | | | |
| 2016 – 2017 | 616 | 144 | 30 | Ref | — | — | Ref | — | — |
| 2008 – 2009 | 553 | 218 | 87 | 0.46 | 0.41 – 0.51 | <0.001 | 0.45 | 0.40 – 0.50 | <0.001 |
| 2010 – 2011 | 555 | 184 | 74 | 0.56 | 0.50 – 0.63 | <0.001 | 0.56 | 0.50 – 0.63 | <0.001 |
| 2012 – 2013 | 580 | 188 | 74 | 0.62 | 0.55 – 0.69 | <0.001 | 0.63 | 0.56 – 0.70 | <0.001 |
| 2014 – 2015 | 687 | 203 | 48 | 0.75 | 0.67 – 0.83 | <0.001 | 0.76 | 0.68 – 0.85 | <0.001 |
| 2018 – 2019 | 458 | 70 | 21 | 1.46 | 1.27 – 1.66 | <0.001 | 1.50 | 1.32 – 1.72 | <0.001 |
| 2020 – 2021 | 297 | 69 | 10 | 1.44 | 1.24 – 1.67 | <0.001 | 1.52 | 1.30 – 1.76 | <0.001 |
| 2022^‡^ | 190 | 90 | 4 | 1.36 | 1.14 – 1.61 | <0.001 | 1.44 | 1.21 – 1.71 | <0.001 |
| **Age at diagnosis in years** | | | | | | | | | |
| 25 – 44 | 2,551 | 697 | 208 | Ref | — | — | Ref | — | — |
| 0 – 24 | 666 | 184 | 22 | 0.95 | 0.88 – 1.02 | 0.2 | 1.05 | 0.97 – 1.14 | 0.3 |
| 45 – 64 | 618 | 252 | 78 | 0.86 | 0.79 – 0.94 | 0.001 | 0.84 | 0.76 – 0.92 | <0.001 |
| ≥65 | 101 | 33 | 40 | 0.85 | 0.67 – 1.07 | 0.2 | 0.70 | 0.55 – 0.90 | 0.005 |
| **Sex** | | | | | | | | | |
| Female | 1,674 | 512 | 132 | Ref | — | — | Ref | — | — |
| Male | 2,262 | 654 | 216 | 1.01 | 0.95 – 1.07 | 0.8 | 1.01 | 0.94 – 1.07 | 0.8 |
| **Region of residence at diagnosis** | | | | | | | | | |
| Oslo | 1,001 | 197 | 87 | Ref | — | — | Ref | — | — |
| Mid Norway | 280 | 78 | 32 | 0.80 | 0.70 – 0.91 | <0.001 | 0.78 | 0.68 – 0.89 | <0.001 |
| Northern Norway | 339 | 132 | 21 | 0.69 | 0.62 – 0.77 | <0.001 | 0.66 | 0.59 – 0.75 | <0.001 |
| South-east Norway (excluding Oslo) | 1,559 | 413 | 106 | 0.91 | 0.84 – 0.99 | 0.021 | 0.87 | 0.80 – 0.94 | <0.001 |
| Western Norway | 757 | 346 | 102 | 0.63 | 0.58 – 0.69 | <0.001 | 0.59 | 0.54 – 0.65 | <0.001 |
| **Place of birth** | | | | | | | | | |
| Norway | 227 | 104 | 57 | Ref | — | — | Ref | — | — |
| Outside Norway | 3,709 | 1,062 | 291 | 1.46 | 1.27 – 1.68 | <0.001 | 1.21 | 1.04 – 1.41 | 0.013 |

*CI: Confidence interval. SHR: Subdistribution-hazard ratio.*

*^†^ Linkage to care is defined as a specialist outpatient consultation or treatment for chronic hepatitis B after diagnosis.*

*Table S13. Relative risk of being retained in care in the last 12 months from univariable and multivariable log-binomial regression, patients diagnosed with chronic hepatitis B still resident at the end of 2022, Norway, 2008 – 2022, Norway, 2008 – 2022, place of birth by region.*

| **Characteristic** | **Retained in care^†^** | | **Univariable** | | | **Multivariable** | | |
| --- | --- | --- | --- | --- | --- | --- | --- | --- |
|  | **Yes** | **No** | **RR** | **95% CI** | **P value** | **RR** | **95% CI** | **P value** |
| **Year of diagnosis^‡^** | | | | | | | | |
| 2016 – 2017 | 652 | 654 | Ref | — | — | Ref | — | — |
| 2008 – 2009 | 808 | 988 | 0.90 | 0.84 – 0.97 | 0.006 | 0.92 | 0.85 – 0.99 | 0.025 |
| 2010 – 2011 | 487 | 737 | 0.80 | 0.73 – 0.87 | <0.001 | 0.82 | 0.75 – 0.89 | <0.001 |
| 2012 – 2013 | 555 | 684 | 0.90 | 0.83 – 0.97 | 0.010 | 0.90 | 0.83 – 0.97 | 0.007 |
| 2014 – 2015 | 656 | 725 | 0.95 | 0.88 – 1.03 | 0.2 | 0.96 | 0.89 – 1.04 | 0.3 |
| 2018 – 2019 | 556 | 386 | 1.18 | 1.10 – 1.28 | <0.001 | 1.15 | 1.07 – 1.24 | <0.001 |
| 2020 – 2021 | 435 | 222 | 1.33 | 1.23 – 1.43 | <0.001 | 1.30 | 1.20 – 1.40 | <0.001 |
| 2022 | 292 | 78 | 1.58 | 1.46 – 1.70 | <0.001 | 1.51 | 1.41 – 1.62 | <0.001 |
| **Age at end of study period in years** | | | | | | | | |
| 25 – 44 | 2,112 | 1,932 | Ref | — | — | Ref | — | — |
| 0 – 24 | 166 | 133 | 1.06 | 0.95 – 1.18 | 0.3 | 0.98 | 0.89 – 1.09 | 0.7 |
| 45 – 64 | 1,817 | 1,931 | 0.93 | 0.89 – 0.97 | <0.001 | 0.97 | 0.94 – 1.01 | 0.2 |
| ≥65 | 346 | 478 | 0.80 | 0.74 – 0.87 | <0.001 | 0.89 | 0.82 – 0.96 | 0.003 |
| **Sex** | | | | | | | | |
| Female | 1,990 | 1,944 | Ref | — | — | Ref | — | — |
| Male | 2,451 | 2,530 | 0.97 | 0.93 – 1.01 | 0.2 | 1.05 | 1.01 – 1.09 | 0.023 |
| **Region of residence at end of study period** | | | | | | | | |
| Oslo | 1,111 | 1,135 | Ref | — | — | Ref | — | — |
| Mid Norway | 314 | 275 | 1.08 | 0.99 – 1.17 | 0.089 | 1.01 | 0.94 – 1.09 | 0.7 |
| Northern Norway | 220 | 214 | 1.02 | 0.92 – 1.13 | 0.6 | 0.93 | 0.84 – 1.02 | 0.13 |
| South-east Norway (excluding Oslo) | 1,952 | 2,019 | 0.99 | 0.94 – 1.05 | 0.8 | 0.97 | 0.93 – 1.02 | 0.3 |
| Western Norway | 844 | 831 | 1.02 | 0.96 – 1.08 | 0.6 | 0.98 | 0.93 – 1.04 | 0.5 |
| **Place of birth^§^** | | | | | | | | |
| Norway | 333 | 793 | Ref | — | — | Ref | — | — |
| North, West and Southern Europe (excluding Norway) | 311 | 282 | 1.77 | 1.58 – 2.00 | <0.001 | 1.60 | 1.42 – 1.79 | <0.001 |
| America | 22 | 27 | 1.52 | 1.05 – 2.02 | 0.011 | 1.43 | 1.05 – 1.95 | 0.024 |
| Central and Eastern Asia | 311 | 194 | 2.08 | 1.86 – 2.33 | <0.001 | 1.96 | 1.76 – 2.18 | <0.001 |
| Eastern Europe | 372 | 386 | 1.66 | 1.48 – 1.86 | <0.001 | 1.51 | 1.35 – 1.70 | <0.001 |
| North Africa and Western Asia | 456 | 437 | 1.73 | 1.55 – 1.93 | <0.001 | 1.59 | 1.42 – 1.77 | <0.001 |
| South-eastern Asia and Oceania | 1,150 | 781 | 2.01 | 1.83 – 2.22 | <0.001 | 1.91 | 1.74 – 2.11 | <0.001 |
| Southern Asia | 353 | 346 | 1.71 | 1.52 – 1.92 | <0.001 | 1.62 | 1.44 – 1.82 | <0.001 |
| Sub-Saharan Africa | 1,133 | 1,228 | 1.62 | 1.47 – 1.80 | <0.001 | 1.53 | 1.38 – 1.69 | <0.001 |

*CI: Confidence interval. RR: Relative risk.*

*^†^ Retention in care is defined as a specialist outpatient consultation for chronic hepatitis B, treatment for chronic hepatitis B or primary care consultation for viral hepatitis.*

*^‡^ For patients identified in NPR (n=4,816), the date of first consultation for chronic hepatitis B is used as a proxy for date of diagnosis. The supplement, part 1, shows that CHB consultation dates in NPR closely reflect diagnosis dates in MSIS. However, one important exception will be 2008 – 2009, as patients identified in NPR for these years will include a notable, yet unknown, proportion who were diagnosed before 2008, and for whom the first consultation for chronic hepatitis B in our dataset reflects a consultation for clinical follow-up.*

*^§^ Categorised according to UN groupings: <https://unstats.un.org/unsd/methodology/m49>.*

# **Data source determining retention in care** **in the last 12 months of the study period**

*Table S14. Data source determining retention in care among patients still resident in Norway at the end of the study period and retained in care in the last 12 months of the study period.*

| **Year of diagnosis** | **Number of patients** | **Data source determining retention in care** | | |
| --- | --- | --- | --- | --- |
|  |  | **NPR** | **Not in NPR but on treatment** | **Not in NPR or on treatment, but in NRPHC** |
| 2008 – 2009 | 813 | 480 (59%) | 246 (30%) | 87 (11%) |
| 2010 – 2011 | 491 | 301 (61%) | 123 (25%) | 67 (14%) |
| 2012 – 2013 | 556 | 353 (63%) | 129 (23%) | 74 (13%) |
| 2014 – 2015 | 660 | 422 (64%) | 141 (21%) | 97 (15%) |
| 2016 – 2017 | 655 | 440 (67%) | 148 (23%) | 67 (10%) |
| 2018 – 2019 | 556 | 389 (70%) | 98 (18%) | 69 (12%) |
| 2020 – 2021 | 437 | 308 (70%) | 84 (19%) | 45 (10%) |
| 2022 | 308 | 220 (71%) | 34 (11%) | 54 (18%) |
| Overall | 4,476 | 2,913 (65%) | 1,003 (22%) | 560 (13%) |

*NPR: Norwegian Patient Registry. NRPHC: Norwegian Registry for Primary Health Care.*

In the sensitivity analysis expanding the definition of retained in care to the last 18 months of the study period (January 2022 – June 2023), the distribution by data source among the 4,946 defined as retained in care was:

- NPR: 3,264 (66%)
- Not in NPR but on treatment: 1,020 (21%)
- Not in NPR or on treatment, but in NRPHC: 662 (13%)

# **Distribution of ATC codes among treated patients**

In Table S15 the number of individuals registered with each ATC code used to define treatment for hepatitis B is presented for patients ever treated and those treated in the last 12 months. The number of individuals is higher than in Table 2 in the manuscript (patients ever treated: 3,509 vs. 2,454; last 12 months: 1,652 vs. 1,528) as some individuals received more than one ATC code in the defined period, predominantly at least one prescription of tenofovir disoproxil fumarate and at least one prescription of entecavir.

In the last 12 months, 119 of the 1,528 treated individuals received a prescription for more than one ATC code. Of these 119, 97 (82%) received at least one prescription for tenofovir disoproxil fumarate and at least one prescription for entecavir. All the 119 received at least one prescription for tenofovir disoproxil fumarate, entecavir and/or tenofovir alafenamide. The three patients registered as having received a prescription for adefovir dipivoxil in the last 12 months also received a prescription for tenofovir disoproxil fumarate, entecavir and/or tenofovir alafenamide in the same period. For the eight patients who received peginterferon in the last 12 months, five also received a prescription for another ATC code in the same period. For three patients, peginterferon was the only ATC code registered.

Among all 2,454 treated patients, 2,129 (87%) received at least one prescription for tenofovir disoproxil fumarate and/or at least one prescription for entecavir. Of the remaining treated patients, 68 (2.8%) received tenofovir alafenamide only, 209 (8.5%) received peginterferon only and 43 (1.8%) received lamivudine only. Five patients were registered with adefovir dipivoxil only and none with telbivudine only.

*Table S15. Number of individuals registered with each ATC code defining treatment for hepatitis B.*

| **Name of medicine** | **ATC code** | **Whole study period** | **Last 12 months of study period** |
| --- | --- | --- | --- |
| Lamivudine | J05AF05 | 133 (3.8%) | 9 (0.5%) |
| Tenofovir disoproxil fumarate | J05AF07 | 1,550 (44%) | 811 (49%) |
| Adefovir dipivoxil | J05AF08 | 45 (1.3%) | 3 (0.2%) |
| Entecavir | J05AF10 | 1,174 (33%) | 734 (44%) |
| Telbivudine | J05AF11 | 14 (0.4%) | 0 (0%) |
| Tenofovir alafenamide | J05AF13 | 204 (5.8%) | 87 (5.3%) |
| Peginterferon alfa-2b | L03AB10 | 65 (1.9%) | 0 (0%) |
| Peginterferon alfa-2a | L03AB11 | 324 (9.2%) | 8 (0.5%) |
